# Aluminum Lined Rubber Plates

**Published:** 1897-12

**Authors:** 


### Aluminum Lined Rubber Plates.

Dr. J. D. Fahnestock of this city, has devised a method of lining rubber plates on the palatal side with aluminum for contact with the mucous membrane in the mouth; this is certainly a great improvement over the rubber alone. The use of this combination is very simple and easy in the hands of any good dentist. It is certainly a very valuable improvement and ought to be in the hands of every dentist who is much engaged in prosthetic work. Dr. Fahnestock will give the needed instruction to any one who wishes to use it.

A MOVEMENT is on foot in Philadelphia to abolish street noises. Dr. Geo. M. Gould is taking an active part in the same.

